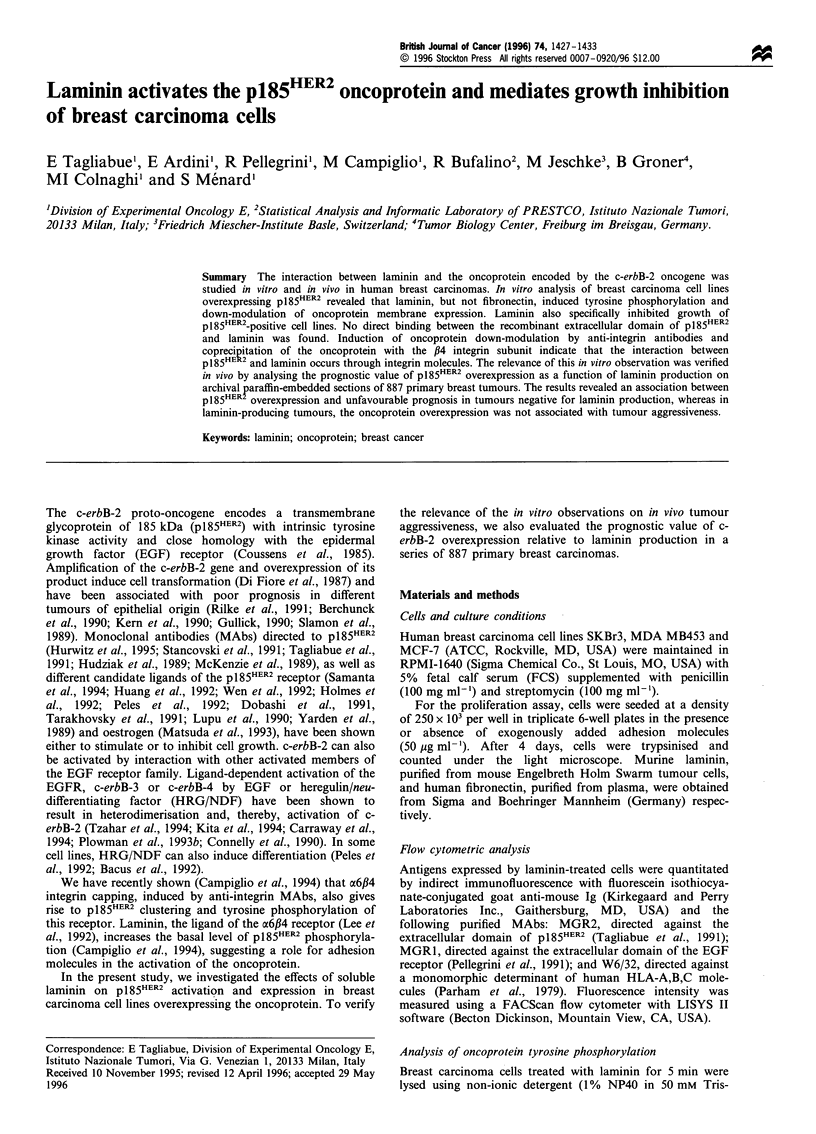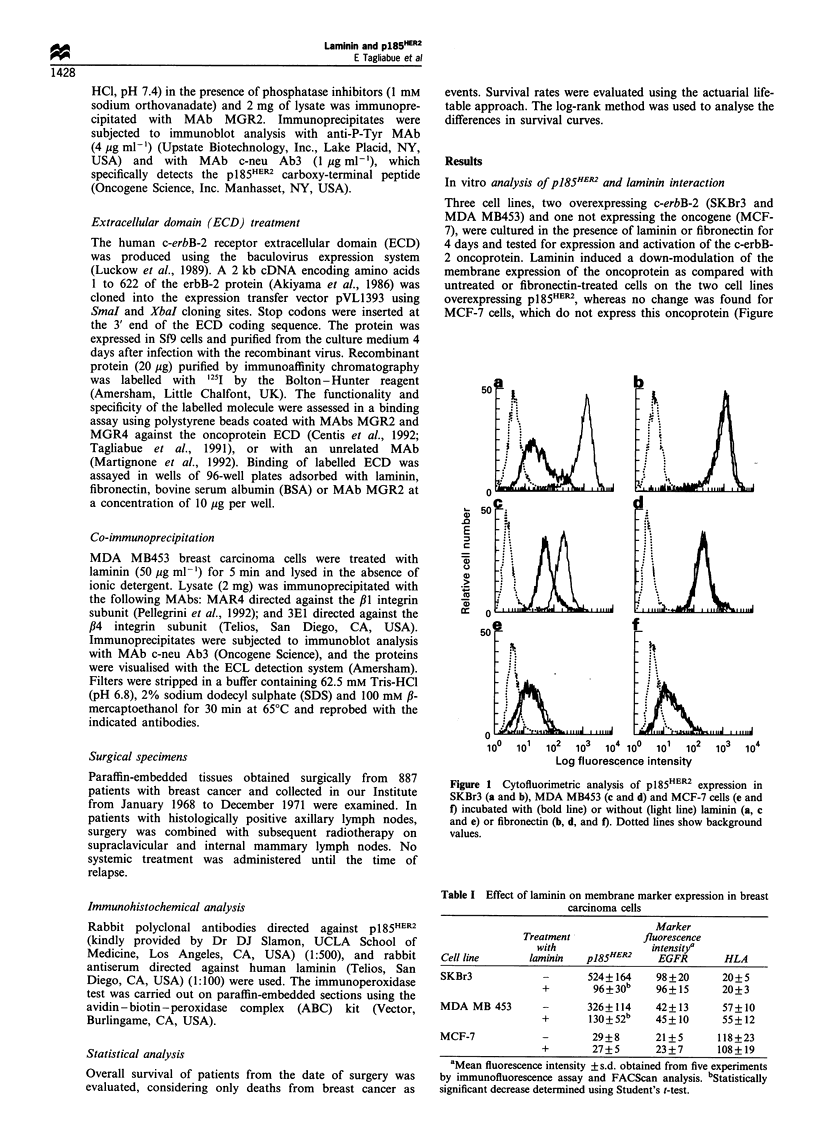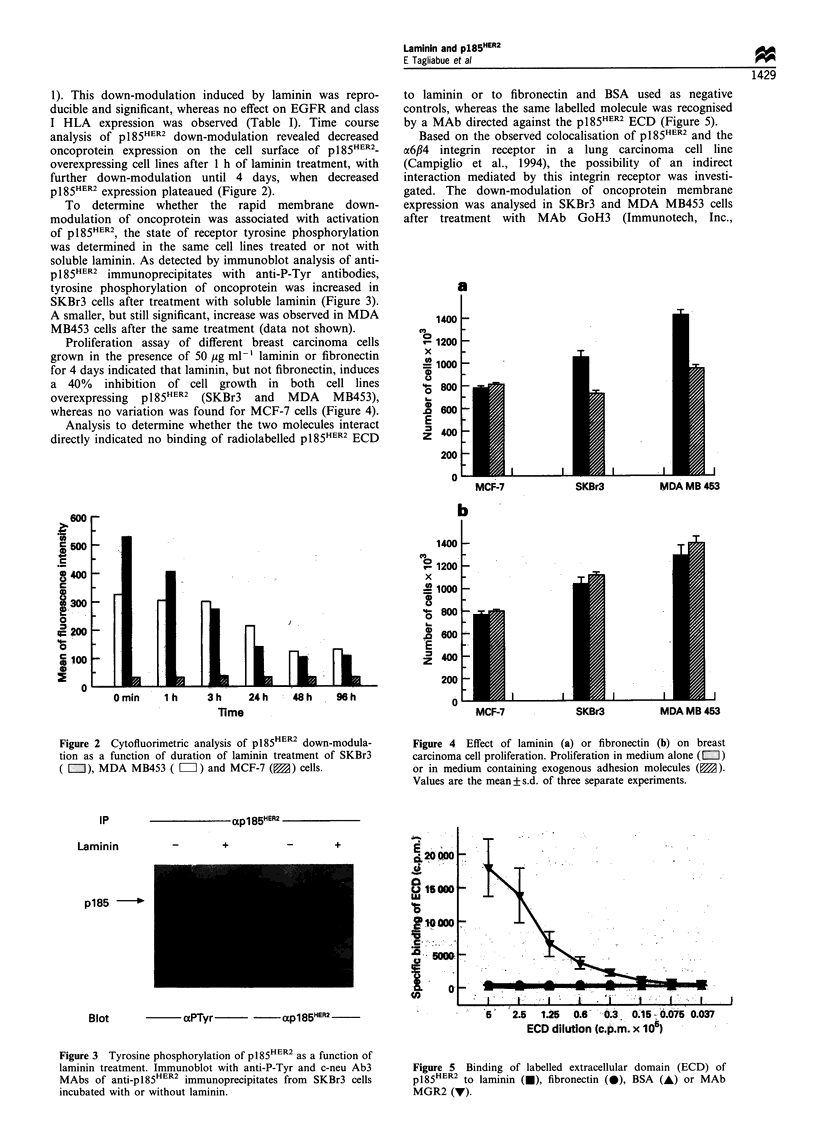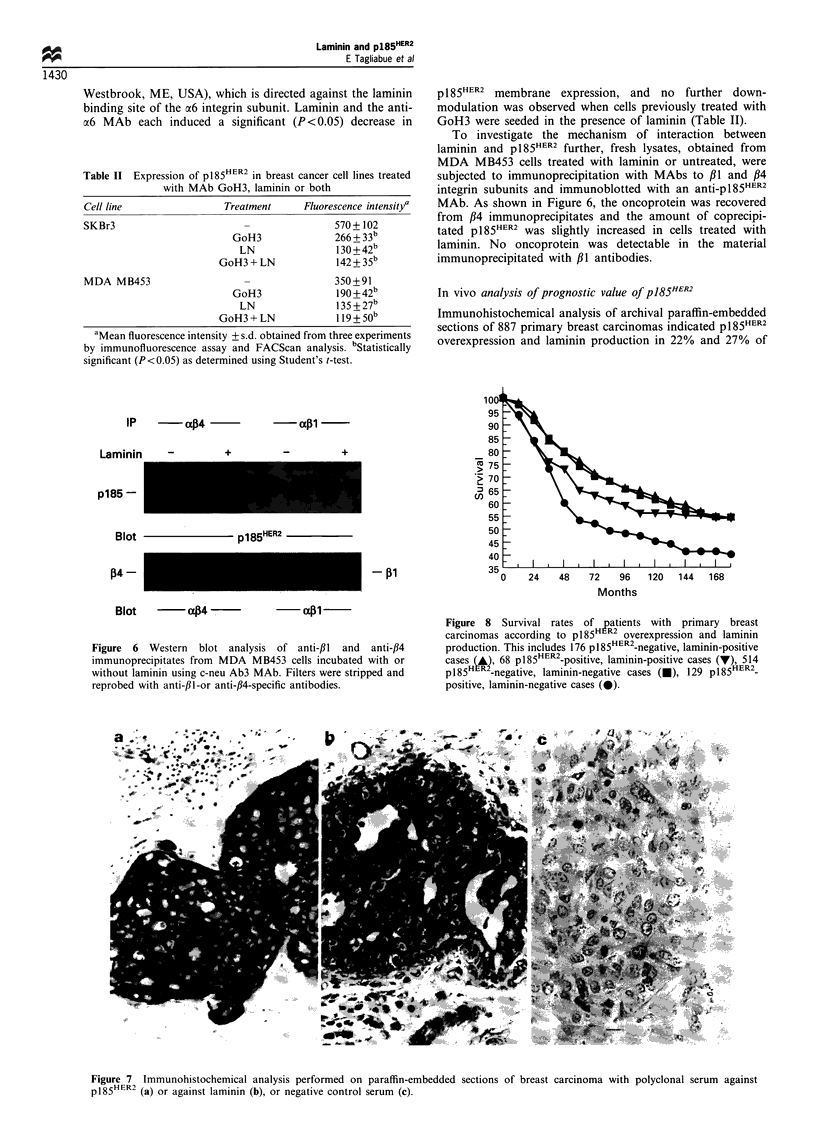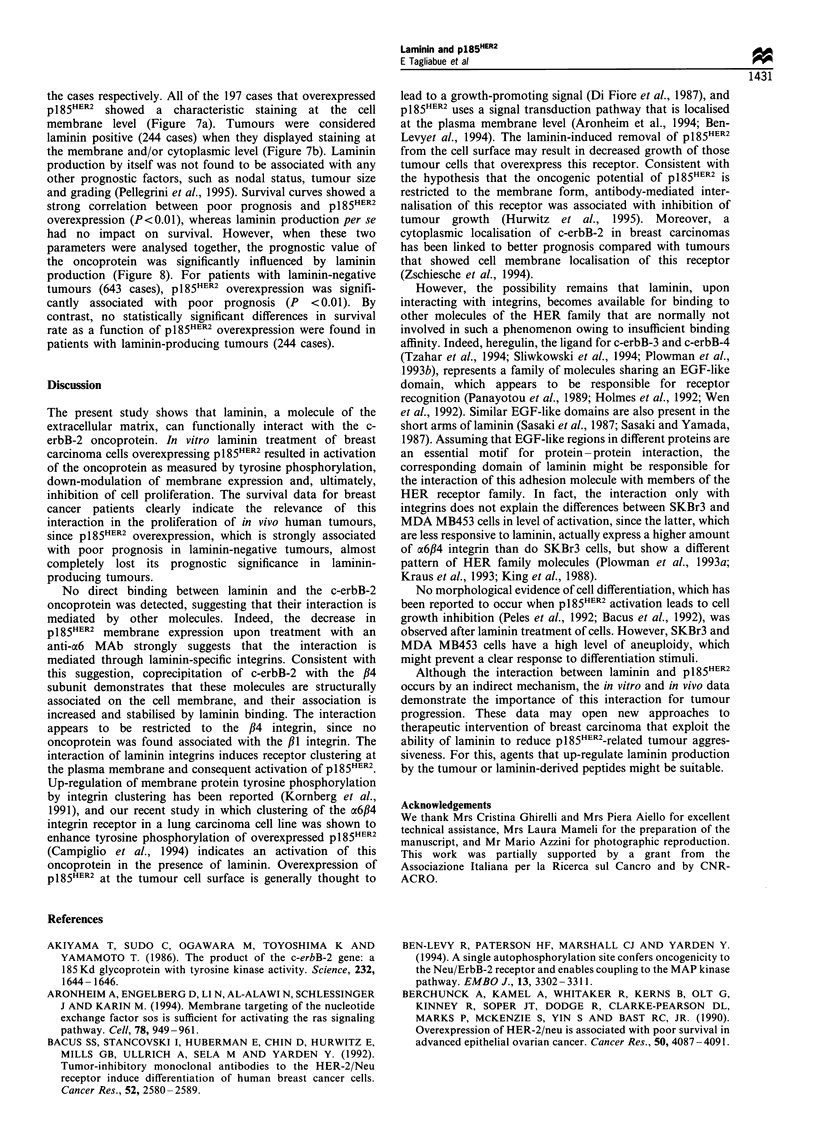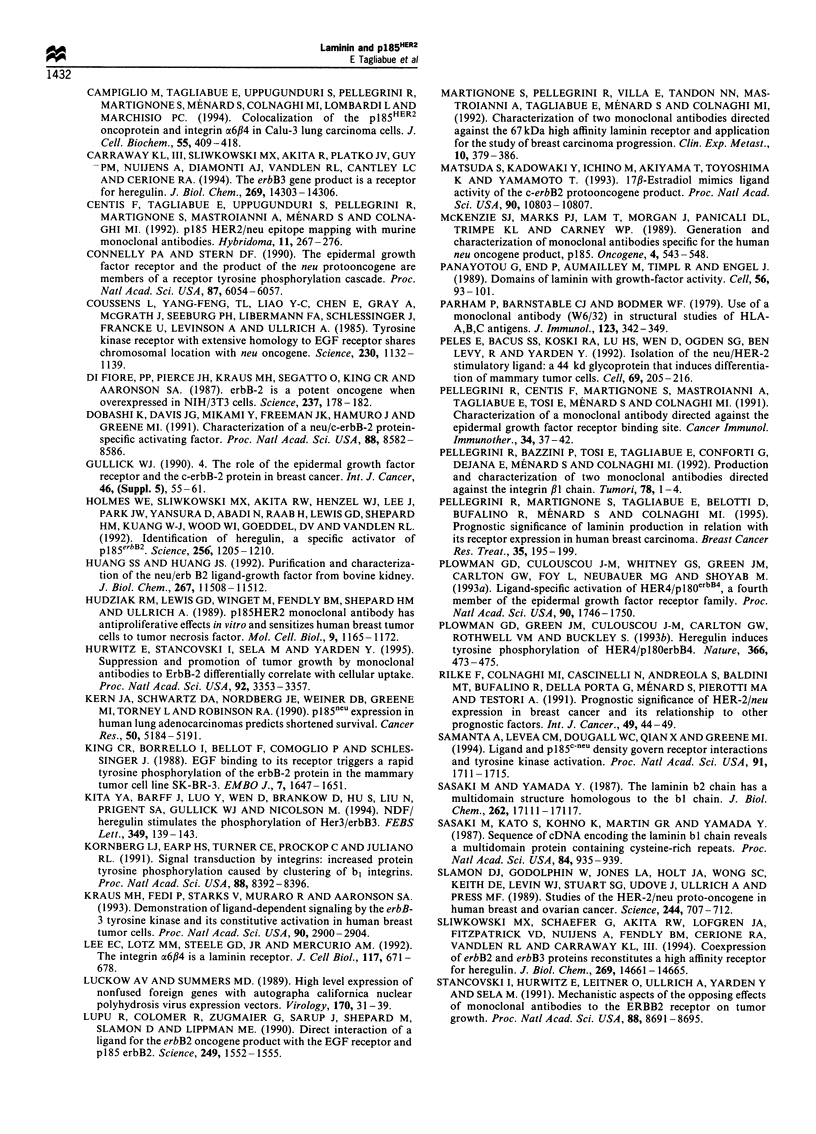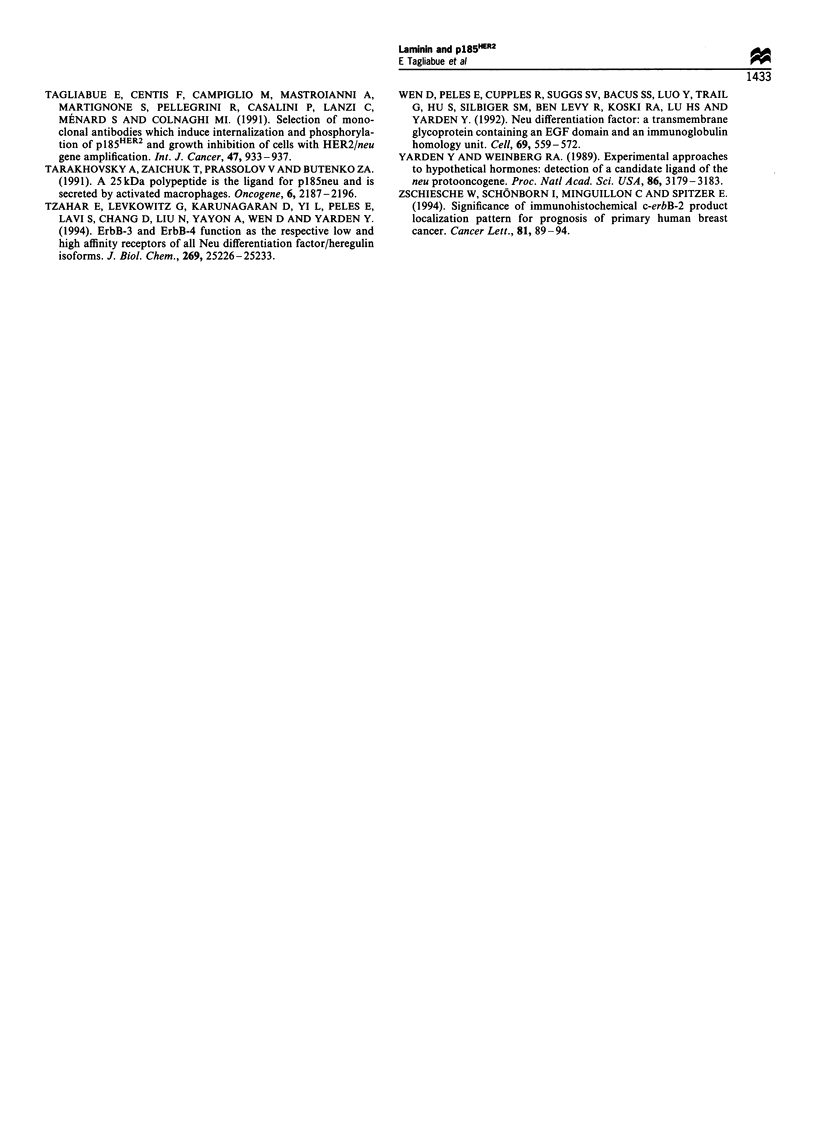# Laminin activates the p185HER2 oncoprotein and mediates growth inhibition of breast carcinoma cells.

**DOI:** 10.1038/bjc.1996.560

**Published:** 1996-11

**Authors:** E. Tagliabue, E. Ardini, R. Pellegrini, M. Campiglio, R. Bufalino, M. Jeschke, B. Groner, M. I. Colnaghi, S. Ménard

**Affiliations:** Division of Experimental Oncology E, Istituto Nazionale Tumori, Milan, Italy.

## Abstract

**Images:**


British Journal of Cancer (1996) 74, 1427–1433
© 1996 Stockton Press All rights reserved 0007–0920/96 $12.00

### Laminin activates the p185$^{HER2}$ oncoprotein and mediates growth inhibition of breast carcinoma cells

E Tagliabue[1], E Ardini[1], R Pellegrini[1], M Campiglio[1], R Bufalino[2], M Jeschke[3], B Groner[4], MI Colnaghi[1] and S Ménard[1]

[1]*Division of Experimental Oncology E,* [2]*Statistical Analysis and Informatic Laboratory of PRESTCO, Istituto Nazionale Tumori, 20133 Milan, Italy;* [3]*Friedrich Miescher-Institute Basle, Switzerland;* [4]*Tumor Biology Center, Freiburg im Breisgau, Germany.*

**Summary** The interaction between laminin and the oncoprotein encoded by the c-*erb*B-2 oncogene was studied *in vitro* and *in vivo* in human breast carcinomas. *In vitro* analysis of breast carcinoma cell lines overexpressing p185$^{HER2}$ revealed that laminin, but not fibronectin, induced tyrosine phosphorylation and down-modulation of oncoprotein membrane expression. Laminin also specifically inhibited growth of p185$^{HER2}$-positive cell lines. No direct binding between the recombinant extracellular domain of p185$^{HER2}$ and laminin was found. Induction of oncoprotein down-modulation by anti-integrin antibodies and coprecipitation of the oncoprotein with the $\beta 4$ integrin subunit indicate that the interaction between p185$^{HER2}$ and laminin occurs through integrin molecules. The relevance of this *in vitro* observation was verified *in vivo* by analysing the prognostic value of p185$^{HER2}$ overexpression as a function of laminin production on archival paraffin-embedded sections of 887 primary breast tumours. The results revealed an association between p185$^{HER2}$ overexpression and unfavourable prognosis in tumours negative for laminin production, whereas in laminin-producing tumours, the oncoprotein overexpression was not associated with tumour aggressiveness.

**Keywords:** laminin; oncoprotein; breast cancer

The c-*erb*B-2 proto-oncogene encodes a transmembrane glycoprotein of 185 kDa (p185$^{HER2}$) with intrinsic tyrosine kinase activity and close homology with the epidermal growth factor (EGF) receptor (Coussens *et al.*, 1985). Amplification of the c-*erb*B-2 gene and overexpression of its product induce cell transformation (Di Fiore *et al.*, 1987) and have been associated with poor prognosis in different tumours of epithelial origin (Rilke *et al.*, 1991; Berchunck *et al.*, 1990; Kern *et al.*, 1990; Gullick, 1990; Slamon *et al.*, 1989). Monoclonal antibodies (MAbs) directed to p185$^{HER2}$ (Hurwitz *et al.*, 1995; Stancovski *et al.*, 1991; Tagliabue *et al.*, 1991; Hudziak *et al.*, 1989; McKenzie *et al.*, 1989), as well as different candidate ligands of the p185$^{HER2}$ receptor (Samanta *et al.*, 1994; Huang *et al.*, 1992; Wen *et al.*, 1992; Holmes *et al.*, 1992; Peles *et al.*, 1992; Dobashi *et al.*, 1991, Tarakhovsky *et al.*, 1991; Lupu *et al.*, 1990; Yarden *et al.*, 1989) and oestrogen (Matsuda *et al.*, 1993), have been shown either to stimulate or to inhibit cell growth. c-*erb*B-2 can also be activated by interaction with other activated members of the EGF receptor family. Ligand-dependent activation of the EGFR, c-*erb*B-3 or c-*erb*B-4 by EGF or heregulin/*neu*-differentiating factor (HRG/NDF) have been shown to result in heterodimerisation and, thereby, activation of c-*erb*B-2 (Tzahar *et al.*, 1994; Kita *et al.*, 1994; Carraway *et al.*, 1994; Plowman *et al.*, 1993*b*; Connelly *et al.*, 1990). In some cell lines, HRG/NDF can also induce differentiation (Peles *et al.*, 1992; Bacus *et al.*, 1992).

We have recently shown (Campiglio *et al.*, 1994) that $\alpha 6\beta 4$ integrin capping, induced by anti-integrin MAbs, also gives rise to p185$^{HER2}$ clustering and tyrosine phosphorylation of this receptor. Laminin, the ligand of the $\alpha 6\beta 4$ receptor (Lee *et al.*, 1992), increases the basal level of p185$^{HER2}$ phosphorylation (Campiglio *et al.*, 1994), suggesting a role for adhesion molecules in the activation of the oncoprotein.

In the present study, we investigated the effects of soluble laminin on p185$^{HER2}$ activation and expression in breast carcinoma cell lines overexpressing the oncoprotein. To verify the relevance of the *in vitro* observations on *in vivo* tumour aggressiveness, we also evaluated the prognostic value of c-*erb*B-2 overexpression relative to laminin production in a series of 887 primary breast carcinomas.

### Materials and methods

### *Cells and culture conditions*

Human breast carcinoma cell lines SKBr3, MDA MB453 and MCF-7 (ATCC, Rockville, MD, USA) were maintained in RPMI-1640 (Sigma Chemical Co., St Louis, MO, USA) with 5% fetal calf serum (FCS) supplemented with penicillin (100 mg ml$^{-1}$) and streptomycin (100 mg ml$^{-1}$).

For the proliferation assay, cells were seeded at a density of $250 \times 10^3$ per well in triplicate 6-well plates in the presence or absence of exogenously added adhesion molecules (50 $\mu$g ml$^{-1}$). After 4 days, cells were trypsinised and counted under the light microscope. Murine laminin, purified from mouse Engelbreth Holm Swarm tumour cells, and human fibronectin, purified from plasma, were obtained from Sigma and Boehringer Mannheim (Germany) respectively.

### *Flow cytometric analysis*

Antigens expressed by laminin-treated cells were quantitated by indirect immunofluorescence with fluorescein isothiocyanate-conjugated goat anti-mouse Ig (Kirkegaard and Perry Laboratories Inc., Gaithersburg, MD, USA) and the following purified MAbs: MGR2, directed against the extracellular domain of p185$^{HER2}$ (Tagliabue *et al.*, 1991); MGR1, directed against the extracellular domain of the EGF receptor (Pellegrini *et al.*, 1991); and W6/32, directed against a monomorphic determinant of human HLA-A,B,C molecules (Parham *et al.*, 1979). Fluorescence intensity was measured using a FACScan flow cytometer with LISYS II software (Becton Dickinson, Mountain View, CA, USA).

### *Analysis of oncoprotein tyrosine phosphorylation*

Breast carcinoma cells treated with laminin for 5 min were lysed using non-ionic detergent (1% NP40 in 50 mM Tris-

Correspondence: E Tagliabue, Division of Experimental Oncology E, Istituto Nazionale Tumori, Via G. Venezian 1, 20133 Milan, Italy
Received 10 November 1995; revised 12 April 1996; accepted 29 May 1996

HCl, pH 7.4) in the presence of phosphatase inhibitors (1 mM sodium orthovanadate) and 2 mg of lysate was immunoprecipitated with MAb MGR2. Immunoprecipitates were subjected to immunoblot analysis with anti-P-Tyr MAb (4 $\mu g\ ml^{-1}$) (Upstate Biotechnology, Inc., Lake Placid, NY, USA) and with MAb c-neu Ab3 (1 $\mu g\ ml^{-1}$), which specifically detects the p185$^{HER2}$ carboxy-terminal peptide (Oncogene Science, Inc. Manhasset, NY, USA).

### *Extracellular domain (ECD) treatment*

The human c-*erb*B-2 receptor extracellular domain (ECD) was produced using the baculovirus expression system (Luckow *et al.*, 1989). A 2 kb cDNA encoding amino acids 1 to 622 of the erbB-2 protein (Akiyama *et al.*, 1986) was cloned into the expression transfer vector pVL1393 using *SmaI* and *XbaI* cloning sites. Stop codons were inserted at the 3′ end of the ECD coding sequence. The protein was expressed in Sf9 cells and purified from the culture medium 4 days after infection with the recombinant virus. Recombinant protein (20 $\mu g$) purified by immunoaffinity chromatography was labelled with $^{125}I$ by the Bolton–Hunter reagent (Amersham, Little Chalfont, UK). The functionality and specificity of the labelled molecule were assessed in a binding assay using polystyrene beads coated with MAbs MGR2 and MGR4 against the oncoprotein ECD (Centis *et al.*, 1992; Tagliabue *et al.*, 1991), or with an unrelated MAb (Martignone *et al.*, 1992). Binding of labelled ECD was assayed in wells of 96-well plates adsorbed with laminin, fibronectin, bovine serum albumin (BSA) or MAb MGR2 at a concentration of 10 $\mu g$ per well.

### *Co-immunoprecipitation*

MDA MB453 breast carcinoma cells were treated with laminin (50 $\mu g\ ml^{-1}$) for 5 min and lysed in the absence of ionic detergent. Lysate (2 mg) was immunoprecipitated with the following MAbs: MAR4 directed against the $\beta$1 integrin subunit (Pellegrini *et al.*, 1992); and 3E1 directed against the $\beta$4 integrin subunit (Telios, San Diego, CA, USA). Immunoprecipitates were subjected to immunoblot analysis with MAb c-neu Ab3 (Oncogene Science), and the proteins were visualised with the ECL detection system (Amersham). Filters were stripped in a buffer containing 62.5 mM Tris-HCl (pH 6.8), 2% sodium dodecyl sulphate (SDS) and 100 mM $\beta$-mercaptoethanol for 30 min at 65°C and reprobed with the indicated antibodies.

### *Surgical specimens*

Paraffin-embedded tissues obtained surgically from 887 patients with breast cancer and collected in our Institute from January 1968 to December 1971 were examined. In patients with histologically positive axillary lymph nodes, surgery was combined with subsequent radiotherapy on supraclavicular and internal mammary lymph nodes. No systemic treatment was administered until the time of relapse.

### *Immunohistochemical analysis*

Rabbit polyclonal antibodies directed against p185$^{HER2}$ (kindly provided by Dr DJ Slamon, UCLA School of Medicine, Los Angeles, CA, USA) (1:500), and rabbit antiserum directed against human laminin (Telios, San Diego, CA, USA) (1:100) were used. The immunoperoxidase test was carried out on paraffin-embedded sections using the avidin–biotin–peroxidase complex (ABC) kit (Vector, Burlingame, CA, USA).

### *Statistical analysis*

Overall survival of patients from the date of surgery was evaluated, considering only deaths from breast cancer as events. Survival rates were evaluated using the actuarial life-table approach. The log-rank method was used to analyse the differences in survival curves.

### Results

### In vitro *analysis of p185$^{HER2}$ and laminin interaction*

Three cell lines, two overexpressing c-*erb*B-2 (SKBr3 and MDA MB453) and one not expressing the oncogene (MCF-7), were cultured in the presence of laminin or fibronectin for 4 days and tested for expression and activation of the c-erbB-2 oncoprotein. Laminin induced a down-modulation of the membrane expression of the oncoprotein as compared with untreated or fibronectin-treated cells on the two cell lines overexpressing p185$^{HER2}$, whereas no change was found for MCF-7 cells, which do not express this oncoprotein (Figure

**Figure 1** Cytofluorimetric analysis of p185$^{HER2}$ expression in SKBr3 (**a** and **b**), MDA MB453 (**c** and **d**) and MCF-7 cells (**e** and **f**) incubated with (bold line) or without (light line) laminin (**a**, **c** and **e**) or fibronectin (**b**, **d**, and **f**). Dotted lines show background values.

**Table I** Effect of laminin on membrane marker expression in breast carcinoma cells

| *Cell line* | *Treatment with laminin* | *p185$^{HER2}$* | *Marker fluorescence intensity$^a$ EGFR* | *HLA* |
|---|---|---|---|---|
| SKBr3 | – | 524±164 | 98±20 | 20±5 |
| | + | 96±30$^b$ | 96±15 | 20±3 |
| MDA MB 453 | – | 326±114 | 42±13 | 57±10 |
| | + | 130±52$^b$ | 45±10 | 55±12 |
| MCF-7 | – | 29±8 | 21±5 | 118±23 |
| | + | 27±5 | 23±7 | 108±19 |

$^a$Mean fluorescence intensity ±s.d. obtained from five experiments by immunofluorescence assay and FACScan analysis. $^b$Statistically significant decrease determined using Student's *t*-test.

1). This down-modulation induced by laminin was reproducible and significant, whereas no effect on EGFR and class I HLA expression was observed (Table I). Time course analysis of p185$^{HER2}$ down-modulation revealed decreased oncoprotein expression on the cell surface of p185$^{HER2}$-overexpressing cell lines after 1 h of laminin treatment, with further down-modulation until 4 days, when decreased p185$^{HER2}$ expression plateaued (Figure 2).

To determine whether the rapid membrane down-modulation of oncoprotein was associated with activation of p185$^{HER2}$, the state of receptor tyrosine phosphorylation was determined in the same cell lines treated or not with soluble laminin. As detected by immunoblot analysis of anti-p185$^{HER2}$ immunoprecipitates with anti-P-Tyr antibodies, tyrosine phosphorylation of oncoprotein was increased in SKBr3 cells after treatment with soluble laminin (Figure 3). A smaller, but still significant, increase was observed in MDA MB453 cells after the same treatment (data not shown).

Proliferation assay of different breast carcinoma cells grown in the presence of 50 $\mu$g ml$^{-1}$ laminin or fibronectin for 4 days indicated that laminin, but not fibronectin, induces a 40% inhibition of cell growth in both cell lines overexpressing p185$^{HER2}$ (SKBr3 and MDA MB453), whereas no variation was found for MCF-7 cells (Figure 4).

Analysis to determine whether the two molecules interact directly indicated no binding of radiolabelled p185$^{HER2}$ ECD to laminin or to fibronectin and BSA used as negative controls, whereas the same labelled molecule was recognised by a MAb directed against the p185$^{HER2}$ ECD (Figure 5).

Based on the observed colocalisation of p185$^{HER2}$ and the $\alpha 6\beta 4$ integrin receptor in a lung carcinoma cell line (Campiglio et al., 1994), the possibility of an indirect interaction mediated by this integrin receptor was investigated. The down-modulation of oncoprotein membrane expression was analysed in SKBr3 and MDA MB453 cells after treatment with MAb GoH3 (Immunotech, Inc.,

**Figure 2** Cytofluorimetric analysis of p185$^{HER2}$ down-modulation as a function of duration of laminin treatment of SKBr3 ( ), MDA MB453 ( ) and MCF-7 ( ) cells.

**Figure 3** Tyrosine phosphorylation of p185$^{HER2}$ as a function of laminin treatment. Immunoblot with anti-P-Tyr and c-neu Ab3 MAbs of anti-p185$^{HER2}$ immunoprecipitates from SKBr3 cells incubated with or without laminin.

**Figure 4** Effect of laminin (**a**) or fibronectin (**b**) on breast carcinoma cell proliferation. Proliferation in medium alone ( ) or in medium containing exogenous adhesion molecules ( ). Values are the mean ± s.d. of three separate experiments.

**Figure 5** Binding of labelled extracellular domain (ECD) of p185$^{HER2}$ to laminin (■), fibronectin (●), BSA (▲) or MAb MGR2 (▼).

Westbrook, ME, USA), which is directed against the laminin binding site of the α6 integrin subunit. Laminin and the anti-α6 MAb each induced a significant ($P<0.05$) decrease in p185$^{HER2}$ membrane expression, and no further down-modulation was observed when cells previously treated with GoH3 were seeded in the presence of laminin (Table II).

**Table II** Expression of p185$^{HER2}$ in breast cancer cell lines treated with MAb GoH3, laminin or both

| *Cell line* | *Treatment* | *Fluorescence intensity*[a] |
|---|---|---|
| SKBr3 | – | 570±102 |
| | GoH3 | 266±33[b] |
| | LN | 130±42[b] |
| | GoH3+LN | 142±35[b] |
| MDA MB453 | – | 350±91 |
| | GoH3 | 190±42[b] |
| | LN | 135±27[b] |
| | GoH3+LN | 119±50[b] |

[a]Mean fluorescence intensity ±s.d. obtained from three experiments by immunofluorescence assay and FACScan analysis. [b]Statistically significant ($P<0.05$) as determined using Student's *t*-test.

To investigate the mechanism of interaction between laminin and p185$^{HER2}$ further, fresh lysates, obtained from MDA MB453 cells treated with laminin or untreated, were subjected to immunoprecipitation with MAbs to β1 and β4 integrin subunits and immunoblotted with an anti-p185$^{HER2}$ MAb. As shown in Figure 6, the oncoprotein was recovered from β4 immunoprecipitates and the amount of coprecipitated p185$^{HER2}$ was slightly increased in cells treated with laminin. No oncoprotein was detectable in the material immunoprecipitated with β1 antibodies.

### In vivo *analysis of prognostic value of p185$^{HER2}$*

Immunohistochemical analysis of archival paraffin-embedded sections of 887 primary breast carcinomas indicated p185$^{HER2}$ overexpression and laminin production in 22% and 27% of

**Figure 6** Western blot analysis of anti-β1 and anti-β4 immunoprecipitates from MDA MB453 cells incubated with or without laminin using c-neu Ab3 MAb. Filters were stripped and reprobed with anti-β1-or anti-β4-specific antibodies.

**Figure 8** Survival rates of patients with primary breast carcinomas according to p185$^{HER2}$ overexpression and laminin production. This includes 176 p185$^{HER2}$-negative, laminin-positive cases (▲), 68 p185$^{HER2}$-positive, laminin-positive cases (▼), 514 p185$^{HER2}$-negative, laminin-negative cases (■), 129 p185$^{HER2}$-positive, laminin-negative cases (●).

**Figure 7** Immunohistochemical analysis performed on paraffin-embedded sections of breast carcinoma with polyclonal serum against p185$^{HER2}$ (**a**) or against laminin (**b**), or negative control serum (**c**).

the cases respectively. All of the 197 cases that overexpressed p185$^{HER2}$ showed a characteristic staining at the cell membrane level (Figure 7a). Tumours were considered laminin positive (244 cases) when they displayed staining at the membrane and/or cytoplasmic level (Figure 7b). Laminin production by itself was not found to be associated with any other prognostic factors, such as nodal status, tumour size and grading (Pellegrini *et al.*, 1995). Survival curves showed a strong correlation between poor prognosis and p185$^{HER2}$ overexpression ($P<0.01$), whereas laminin production *per se* had no impact on survival. However, when these two parameters were analysed together, the prognostic value of the oncoprotein was significantly influenced by laminin production (Figure 8). For patients with laminin-negative tumours (643 cases), p185$^{HER2}$ overexpression was significantly associated with poor prognosis ($P<0.01$). By contrast, no statistically significant differences in survival rate as a function of p185$^{HER2}$ overexpression were found in patients with laminin-producing tumours (244 cases).

### Discussion

The present study shows that laminin, a molecule of the extracellular matrix, can functionally interact with the c-erbB-2 oncoprotein. *In vitro* laminin treatment of breast carcinoma cells overexpressing p185$^{HER2}$ resulted in activation of the oncoprotein as measured by tyrosine phosphorylation, down-modulation of membrane expression and, ultimately, inhibition of cell proliferation. The survival data for breast cancer patients clearly indicate the relevance of this interaction in the proliferation of *in vivo* human tumours, since p185$^{HER2}$ overexpression, which is strongly associated with poor prognosis in laminin-negative tumours, almost completely lost its prognostic significance in laminin-producing tumours.

No direct binding between laminin and the c-erbB-2 oncoprotein was detected, suggesting that their interaction is mediated by other molecules. Indeed, the decrease in p185$^{HER2}$ membrane expression upon treatment with an anti-$\alpha6$ MAb strongly suggests that the interaction is mediated through laminin-specific integrins. Consistent with this suggestion, coprecipitation of c-erbB-2 with the $\beta4$ subunit demonstrates that these molecules are structurally associated on the cell membrane, and their association is increased and stabilised by laminin binding. The interaction appears to be restricted to the $\beta4$ integrin, since no oncoprotein was found associated with the $\beta1$ integrin. The interaction of laminin integrins induces receptor clustering at the plasma membrane and consequent activation of p185$^{HER2}$. Up-regulation of membrane protein tyrosine phosphorylation by integrin clustering has been reported (Kornberg *et al.*, 1991), and our recent study in which clustering of the $\alpha6\beta4$ integrin receptor in a lung carcinoma cell line was shown to enhance tyrosine phosphorylation of overexpressed p185$^{HER2}$ (Campiglio *et al.*, 1994) indicates an activation of this oncoprotein in the presence of laminin. Overexpression of p185$^{HER2}$ at the tumour cell surface is generally thought to lead to a growth-promoting signal (Di Fiore *et al.*, 1987), and p185$^{HER2}$ uses a signal transduction pathway that is localised at the plasma membrane level (Aronheim et al., 1994; Ben-Levy*et al.*, 1994). The laminin-induced removal of p185$^{HER2}$ from the cell surface may result in decreased growth of those tumour cells that overexpress this receptor. Consistent with the hypothesis that the oncogenic potential of p185$^{HER2}$ is restricted to the membrane form, antibody-mediated internalisation of this receptor was associated with inhibition of tumour growth (Hurwitz *et al.*, 1995). Moreover, a cytoplasmic localisation of c-erbB-2 in breast carcinomas has been linked to better prognosis compared with tumours that showed cell membrane localisation of this receptor (Zschiesche *et al.*, 1994).

However, the possibility remains that laminin, upon interacting with integrins, becomes available for binding to other molecules of the HER family that are normally not involved in such a phenomenon owing to insufficient binding affinity. Indeed, heregulin, the ligand for c-erbB-3 and c-erbB-4 (Tzahar *et al.*, 1994; Sliwkowski *et al.*, 1994; Plowman *et al.*, 1993*b*), represents a family of molecules sharing an EGF-like domain, which appears to be responsible for receptor recognition (Panayotou *et al.*, 1989; Holmes *et al.*, 1992; Wen *et al.*, 1992). Similar EGF-like domains are also present in the short arms of laminin (Sasaki *et al.*, 1987; Sasaki and Yamada, 1987). Assuming that EGF-like regions in different proteins are an essential motif for protein–protein interaction, the corresponding domain of laminin might be responsible for the interaction of this adhesion molecule with members of the HER receptor family. In fact, the interaction only with integrins does not explain the differences between SKBr3 and MDA MB453 cells in level of activation, since the latter, which are less responsive to laminin, actually express a higher amount of $\alpha6\beta4$ integrin than do SKBr3 cells, but show a different pattern of HER family molecules (Plowman *et al.*, 1993*a*; Kraus *et al.*, 1993; King *et al.*, 1988).

No morphological evidence of cell differentiation, which has been reported to occur when p185$^{HER2}$ activation leads to cell growth inhibition (Peles *et al.*, 1992; Bacus *et al.*, 1992), was observed after laminin treatment of cells. However, SKBr3 and MDA MB453 cells have a high level of aneuploidy, which might prevent a clear response to differentiation stimuli.

Although the interaction between laminin and p185$^{HER2}$ occurs by an indirect mechanism, the *in vitro* and *in vivo* data demonstrate the importance of this interaction for tumour progression. These data may open new approaches to therapeutic intervention of breast carcinoma that exploit the ability of laminin to reduce p185$^{HER2}$-related tumour aggressiveness. For this, agents that up-regulate laminin production by the tumour or laminin-derived peptides might be suitable.

**Acknowledgements**
We thank Mrs Cristina Ghirelli and Mrs Piera Aiello for excellent technical assistance, Mrs Laura Mameli for the preparation of the manuscript, and Mr Mario Azzini for photographic reproduction. This work was partially supported by a grant from the Associazione Italiana per la Ricerca sul Cancro and by CNR-ACRO.

### References

AKIYAMA T, SUDO C, OGAWARA M, TOYOSHIMA K AND YAMAMOTO T. (1986). The product of the c-*erb*B-2 gene: a 185 Kd glycoprotein with tyrosine kinase activity. *Science*, **232**, 1644–1646.

ARONHEIM A, ENGELBERG D, LI N, AL-ALAWI N, SCHLESSINGER J AND KARIN M. (1994). Membrane targeting of the nucleotide exchange factor sos is sufficient for activating the ras signaling pathway. *Cell*, **78**, 949–961.

BACUS SS, STANCOVSKI I, HUBERMAN E, CHIN D, HURWITZ E, MILLS GB, ULLRICH A, SELA M AND YARDEN Y. (1992). Tumor-inhibitory monoclonal antibodies to the HER-2/Neu receptor induce differentiation of human breast cancer cells. *Cancer Res.*, **52**, 2580–2589.

BEN-LEVY R, PATERSON HF, MARSHALL CJ AND YARDEN Y. (1994). A single autophosphorylation site confers oncogenicity to the Neu/ErbB-2 receptor and enables coupling to the MAP kinase pathway. *EMBO J.*, **13**, 3302–3311.

BERCHUNCK A, KAMEL A, WHITAKER R, KERNS B, OLT G, KINNEY R, SOPER JT, DODGE R, CLARKE-PEARSON DL, MARKS P, MCKENZIE S, YIN S AND BAST RC, JR. (1990). Overexpression of HER-2/neu is associated with poor survival in advanced epithelial ovarian cancer. *Cancer Res.*, **50**, 4087–4091.

CAMPIGLIO M, TAGLIABUE E, UPPUGUNDURI S, PELLEGRINI R, MARTIGNONE S, MÉNARD S, COLNAGHI MI, LOMBARDI L AND MARCHISIO PC. (1994). Colocalization of the p185$^{HER2}$ oncoprotein and integrin α6β4 in Calu-3 lung carcinoma cells. *J. Cell. Biochem.*, **55**, 409–418.

CARRAWAY KL, III, SLIWKOWSKI MX, AKITA R, PLATKO JV, GUY PM, NUIJENS A, DIAMONTI AJ, VANDLEN RL, CANTLEY LC AND CERIONE RA. (1994). The *erb*B3 gene product is a receptor for heregulin. *J. Biol. Chem.*, **269**, 14303–14306.

CENTIS F, TAGLIABUE E, UPPUGUNDURI S, PELLEGRINI R, MARTIGNONE S, MASTROIANNI A, MÉNARD S AND COLNAGHI MI. (1992). p185 HER2/neu epitope mapping with murine monoclonal antibodies. *Hybridoma*, **11**, 267–276.

CONNELLY PA AND STERN DF. (1990). The epidermal growth factor receptor and the product of the *neu* protooncogene are members of a receptor tyrosine phosphorylation cascade. *Proc. Natl Acad. Sci. USA*, **87**, 6054–6057.

COUSSENS L, YANG-FENG, TL, LIAO Y-C, CHEN E, GRAY A, MCGRATH J, SEEBURG PH, LIBERMANN FA, SCHLESSINGER J, FRANCKE U, LEVINSON A AND ULLRICH A. (1985). Tyrosine kinase receptor with extensive homology to EGF receptor shares chromosomal location with *neu* oncogene. *Science*, **230**, 1132–1139.

DI FIORE, PP, PIERCE JH, KRAUS MH, SEGATTO O, KING CR AND AARONSON SA. (1987). erbB-2 is a potent oncogene when overexpressed in NIH/3T3 cells. *Science*, **237**, 178–182.

DOBASHI K, DAVIS JG, MIKAMI Y, FREEMAN JK, HAMURO J AND GREENE MI. (1991). Characterization of a neu/c-erbB-2 protein-specific activating factor. *Proc. Natl Acad. Sci. USA*, **88**, 8582–8586.

GULLICK WJ. (1990). 4. The role of the epidermal growth factor receptor and the c-erbB-2 protein in breast cancer. *Int. J. Cancer*, **46, (Suppl. 5)**, 55–61.

HOLMES WE, SLIWKOWSKI MX, AKITA RW, HENZEL WJ, LEE J, PARK JW, YANSURA D, ABADI N, RAAB H, LEWIS GD, SHEPARD HM, KUANG W-J, WOOD WI, GOEDDEL, DV AND VANDLEN RL. (1992). Identification of heregulin, a specific activator of p185$^{erbB2}$. *Science*, **256**, 1205–1210.

HUANG SS AND HUANG JS. (1992). Purification and characterization of the neu/erb B2 ligand-growth factor from bovine kidney. *J. Biol. Chem.*, **267**, 11508–11512.

HUDZIAK RM, LEWIS GD, WINGET M, FENDLY BM, SHEPARD HM AND ULLRICH A. (1989). p185HER2 monoclonal antibody has antiproliferative effects *in vitro* and sensitizes human breast tumor cells to tumor necrosis factor. *Mol. Cell. Biol.*, **9**, 1165–1172.

HURWITZ E, STANCOVSKI I, SELA M AND YARDEN Y. (1995). Suppression and promotion of tumor growth by monoclonal antibodies to ErbB-2 differentially correlate with cellular uptake. *Proc. Natl Acad. Sci. USA*, **92**, 3353–3357.

KERN JA, SCHWARTZ DA, NORDBERG JE, WEINER DB, GREENE MI, TORNEY L AND ROBINSON RA. (1990). p185$^{neu}$ expression in human lung adenocarcinomas predicts shortened survival. *Cancer Res.*, **50**, 5184–5191.

KING CR, BORRELLO I, BELLOT F, COMOGLIO P AND SCHLESSINGER J. (1988). EGF binding to its receptor triggers a rapid tyrosine phosphorylation of the erbB-2 protein in the mammary tumor cell line SK-BR-3. *EMBO J.*, **7**, 1647–1651.

KITA YA, BARFF J, LUO Y, WEN D, BRANKOW D, HU S, LIU N, PRIGENT SA, GULLICK WJ AND NICOLSON M. (1994). NDF/heregulin stimulates the phosphorylation of Her3/erbB3. *FEBS Lett.*, **349**, 139–143.

KORNBERG LJ, EARP HS, TURNER CE, PROCKOP C AND JULIANO RL. (1991). Signal transduction by integrins: increased protein tyrosine phosphorylation caused by clustering of $b_1$ integrins. *Proc. Natl Acad. Sci. USA*, **88**, 8392–8396.

KRAUS MH, FEDI P, STARKS V, MURARO R AND AARONSON SA. (1993). Demonstration of ligand-dependent signaling by the *erbB-3* tyrosine kinase and its constitutive activation in human breast tumor cells. *Proc. Natl Acad. Sci. USA*, **90**, 2900–2904.

LEE EC, LOTZ MM, STEELE GD, JR AND MERCURIO AM. (1992). The integrin α6β4 is a laminin receptor. *J. Cell Biol.*, **117**, 671–678.

LUCKOW AV AND SUMMERS MD. (1989). High level expression of nonfused foreign genes with autographa californica nuclear polyhydrosis virus expression vectors. *Virology*, **170**, 31–39.

LUPU R, COLOMER R, ZUGMAIER G, SARUP J, SHEPARD M, SLAMON D AND LIPPMAN ME. (1990). Direct interaction of a ligand for the *erb*B2 oncogene product with the EGF receptor and p185 erbB2. *Science*, **249**, 1552–1555.

MARTIGNONE S, PELLEGRINI R, VILLA E, TANDON NN, MASTROIANNI A, TAGLIABUE E, MÉNARD S AND COLNAGHI MI, (1992). Characterization of two monoclonal antibodies directed against the 67 kDa high affinity laminin receptor and application for the study of breast carcinoma progression. *Clin. Exp. Metast.*, **10**, 379–386.

MATSUDA S, KADOWAKI Y, ICHINO M, AKIYAMA T, TOYOSHIMA K AND YAMAMOTO T. (1993). 17β-Estradiol mimics ligand activity of the c-*erb*B2 protooncogene product. *Proc. Natl Acad. Sci. USA*, **90**, 10803–10807.

McKENZIE SJ, MARKS PJ, LAM T, MORGAN J, PANICALI DL, TRIMPE KL AND CARNEY WP. (1989). Generation and characterization of monoclonal antibodies specific for the human *neu* oncogene product, p185. *Oncogene*, **4**, 543–548.

PANAYOTOU G, END P, AUMAILLEY M, TIMPL R AND ENGEL J. (1989). Domains of laminin with growth-factor activity. *Cell*, **56**, 93–101.

PARHAM P, BARNSTABLE CJ AND BODMER WF. (1979). Use of a monoclonal antibody (W6/32) in structural studies of HLA-A,B,C antigens. *J. Immunol.*, **123**, 342–349.

PELES E, BACUS SS, KOSKI RA, LU HS, WEN D, OGDEN SG, BEN LEVY, R AND YARDEN Y. (1992). Isolation of the neu/HER-2 stimulatory ligand: a 44 kd glycoprotein that induces differentiation of mammary tumor cells. *Cell*, **69**, 205–216.

PELLEGRINI R, CENTIS F, MARTIGNONE S, MASTROIANNI A, TAGLIABUE E, TOSI E, MÉNARD S AND COLNAGHI MI. (1991). Characterization of a monoclonal antibody directed against the epidermal growth factor receptor binding site. *Cancer Immunol. Immunother.*, **34**, 37–42.

PELLEGRINI R, BAZZINI P, TOSI E, TAGLIABUE E, CONFORTI G, DEJANA E, MÉNARD S AND COLNAGHI MI. (1992). Production and characterization of two monoclonal antibodies directed against the integrin β1 chain. *Tumori*, **78**, 1–4.

PELLEGRINI R, MARTIGNONE S, TAGLIABUE E, BELOTTI D, BUFALINO R, MÉNARD S AND COLNAGHI MI. (1995). Prognostic significance of laminin production in relation with its receptor expression in human breast carcinoma. *Breast Cancer Res. Treat.*, **35**, 195–199.

PLOWMAN GD, CULOUSCOU J-M, WHITNEY GS, GREEN JM, CARLTON GW, FOY L, NEUBAUER MG AND SHOYAB M. (1993*a*). Ligand-specific activation of HER4/p180$^{erbB4}$, a fourth member of the epidermal growth factor receptor family. *Proc. Natl Acad. Sci. USA*, **90**, 1746–1750.

PLOWMAN GD, GREEN JM, CULOUSCOU J-M, CARLTON GW, ROTHWELL VM AND BUCKLEY S. (1993*b*). Heregulin induces tyrosine phosphorylation of HER4/p180erbB4. *Nature*, **366**, 473–475.

RILKE F, COLNAGHI MI, CASCINELLI N, ANDREOLA S, BALDINI MT, BUFALINO R, DELLA PORTA G, MÉNARD S, PIEROTTI MA AND TESTORI A. (1991). Prognostic significance of HER-2/*neu* expression in breast cancer and its relationship to other prognostic factors. *Int. J. Cancer.*, **49**, 44–49.

SAMANTA A, LEVEA CM, DOUGALL WC, QIAN X AND GREENE MI. (1994). Ligand and p185$^{c\text{-}neu}$ density govern receptor interactions and tyrosine kinase activation. *Proc. Natl Acad. Sci. USA*, **91**, 1711–1715.

SASAKI M AND YAMADA Y. (1987). The laminin b2 chain has a multidomain structure homologous to the b1 chain. *J. Biol. Chem.*, **262**, 17111–17117.

SASAKI M, KATO S, KOHNO K, MARTIN GR AND YAMADA Y. (1987). Sequence of cDNA encoding the laminin b1 chain reveals a multidomain protein containing cysteine-rich repeats. *Proc. Natl Acad. Sci. USA*, **84**, 935–939.

SLAMON DJ, GODOLPHIN W, JONES LA, HOLT JA, WONG SC, KEITH DE, LEVIN WJ, STUART SG, UDOVE J, ULLRICH A AND PRESS MF. (1989). Studies of the HER-2/neu proto-oncogene in human breast and ovarian cancer. *Science*, **244**, 707–712.

SLIWKOWSKI MX, SCHAEFER G, AKITA RW, LOFGREN JA, FITZPATRICK VD, NUIJENS A, FENDLY BM, CERIONE RA, VANDLEN RL AND CARRAWAY KL, III. (1994). Coexpression of *erb*B2 and *erb*B3 proteins reconstitutes a high affinity receptor for heregulin. *J. Biol. Chem.*, **269**, 14661–14665.

STANCOVSKI I, HURWITZ E, LEITNER O, ULLRICH A, YARDEN Y AND SELA M. (1991). Mechanistic aspects of the opposing effects of monoclonal antibodies to the ERBB2 receptor on tumor growth. *Proc. Natl Acad. Sci. USA*, **88**, 8691–8695.

TAGLIABUE E, CENTIS F, CAMPIGLIO M, MASTROIANNI A, MARTIGNONE S, PELLEGRINI R, CASALINI P, LANZI C, MÉNARD S AND COLNAGHI MI. (1991). Selection of monoclonal antibodies which induce internalization and phosphorylation of p185$^{HER2}$ and growth inhibition of cells with HER2/*neu* gene amplification. *Int. J. Cancer*, **47**, 933-937.

TARAKHOVSKY A, ZAICHUK T, PRASSOLOV V AND BUTENKO ZA. (1991). A 25 kDa polypeptide is the ligand for p185neu and is secreted by activated macrophages. *Oncogene*, **6**, 2187-2196.

TZAHAR E, LEVKOWITZ G, KARUNAGARAN D, YI L, PELES E, LAVI S, CHANG D, LIU N, YAYON A, WEN D AND YARDEN Y. (1994). ErbB-3 and ErbB-4 function as the respective low and high affinity receptors of all Neu differentiation factor/heregulin isoforms. *J. Biol. Chem.*, **269**, 25226-25233.

WEN D, PELES E, CUPPLES R, SUGGS SV, BACUS SS, LUO Y, TRAIL G, HU S, SILBIGER SM, BEN LEVY R, KOSKI RA, LU HS AND YARDEN Y. (1992). Neu differentiation factor: a transmembrane glycoprotein containing an EGF domain and an immunoglobulin homology unit. *Cell*, **69**, 559-572.

YARDEN Y AND WEINBERG RA. (1989). Experimental approaches to hypothetical hormones: detection of a candidate ligand of the *neu* protooncogene. *Proc. Natl Acad. Sci. USA*, **86**, 3179-3183.

ZSCHIESCHE W, SCHÖNBORN I, MINGUILLON C AND SPITZER E. (1994). Significance of immunohistochemical c-*erb*B-2 product localization pattern for prognosis of primary human breast cancer. *Cancer Lett.*, **81**, 89-94.